# Giant Cell Tumor of the Sacrum: A Narrative Review of Management Challenges and Modalities

**DOI:** 10.3390/healthcare14101381

**Published:** 2026-05-18

**Authors:** Abdulrahman Alaseem, Zyad A. Aldosari, Abdulmalik A. Alduraibi, Rola K. Alzahrani, Abdulaziz S. AlNahari, Motaz AlAqeel, Ibrahim Alshaygy

**Affiliations:** 1 Department of Orthopedic Surgery, College of Medicine, King Saud University, Riyadh 11461, Saudi Arabia; 2 College of Medicine, King Saud University, Riyadh 11461, Saudi Arabia; abdulmalik.alduraibi@gmail.com (A.A.A.); rola.k.alzahrani@gmail.com (R.K.A.)

**Keywords:** denosumab, giant cell tumor of bone, sacral neoplasms, sacrum, selective arterial embolization

## Abstract

**Background/Objectives:** Sacral giant cell tumor of bone (GCTB) is a rare, mostly benign but locally aggressive neoplasm that carries significant diagnostic and treatment challenges due to its anatomic complexity, proximity to sacral nerve roots as well as the blood vessels, and potential impact on bowel, bladder, sexual, and lumbopelvic function and stability. This narrative review aimed to synthesize current evidence on the epidemiology, clinical presentation, diagnostic evaluation, classification, management strategies, outcomes, and surveillance of sacral GCTB. **Methods:** A focused literature search of PubMed/MEDLINE and Google Scholar was conducted for studies published between January 2000 and January 2026, with additional manual review of reference lists. Given the rarity of the tumor and the observed heterogeneity in study designs, treatment strategies, and outcomes, the evidence was synthesized narratively. **Results:** Sacral GCTB mainly affects young adults with an indolent nature, often presenting late with progressive low back or buttock pain, radiculopathy, or neurological deficits. Magnetic resonance imaging is the preferred modality for determining local extent, whereas histopathologic biopsy and molecular testing remain essential for definitive diagnosis. Conventional grading systems, such as Enneking and Campanacci, have limited value in sacral disease, as anatomical extent and anticipated neurological morbidity are more relevant for treatment planning. Surgery remains the cornerstone for resectable disease, yet management plans should balance local tumor control against preservation of sacral nerve roots and mechanical stability. Denosumab, selective arterial embolization, and radiotherapy may play important roles in selected unresectable or high-morbidity cases. Local recurrence remains a major concern, and long-term surveillance is recommended because tumor relapse, treatment-related morbidity, and distant metastasis may occur late. **Conclusions:** Current evidence supports a multidisciplinary, individualized approach to sacral GCTB, guided by tumor extent, expected neurological morbidity, and patient-centered functional outcomes. Prospective multicenter studies are needed to refine treatment algorithms and improve risk stratification.

## 1. Introduction

Giant cell tumor of bone (GCTB) is a benign but locally aggressive neoplasm with low metastatic potential. It typically arises in the epiphysis of long bones in young adults and shows a slight female predominance. GCTB accounts for 4–10% of primary bone tumors, and up to 20% of benign bone tumors. Its pathophysiology is associated with activation of the RANK/RANKL pathway, which promotes osteoclast-like giant cell formation and bone resorption [1]. 

Sacral and axial involvement are uncommon compared with appendicular disease but pose distinct diagnostic and therapeutic challenges. A published series reported the sacrum as the fourth most common site of GCTB, accounting for 1.8–8.2% of cases [2]. In a large single-institution cohort of 1385 primary sacral tumors and tumor-like lesions, sacral GCTB was the most common benign histologic subtype, comprising 205 cases; affected patients had a mean age of 34.4 years and a slight female predominance [3]. Because of their deep pelvic location, insidious onset, and nonspecific early symptoms, these tumors are often diagnosed late, frequently after the development of pain, radicular symptoms, or neurological deficits [3,4,5]. 

Management of sacral GCTB is particularly complex because tumor control must be balanced against preservation of sacral nerve roots, bowel and bladder function, sexual function, and lumbopelvic stability. These anatomical constraints may limit the feasibility of complete excision, increase surgical morbidity, and contribute to higher local recurrence rates than those typically reported for long-bone GCTB [2,6]. In addition, the available literature remains limited by the rarity of the disease and consists largely of retrospective series, small cohorts, and selected case reports, making treatment recommendations difficult to standardize [2,6].

In this context, an updated synthesis of sacral GCTB is needed because contemporary management increasingly extends beyond conventional surgery alone. Recent developments in denosumab-era treatment, molecular diagnostic confirmation, selective arterial embolization, recurrence-related metastatic risk, functional outcome assessment, and long-term surveillance have changed how these tumors are evaluated and managed. This narrative review therefore aims to integrate these evolving areas with sacral-specific anatomical and neurological considerations, emphasizing the balance between local tumor control, nerve-root preservation, treatment-related morbidity, and patient-centered functional outcomes.

## 2. Methodology

This narrative review was developed according to the Scale for the Assessment of Narrative Review Articles (SANRA), which was used to guide the clarity of the review aim, description of the literature search, referencing, scientific reasoning, presentation of evidence, and discussion of limitations [7]. A focused literature search was conducted in PubMed/MEDLINE and Google Scholar for articles published between January 2000 and January 2026. The final search was performed on 16 January 2026. The search was restricted to English-language articles and Articles available in English translation.

A structured search strategy was developed using combinations of keywords and Boolean operators. The core search terms included (“giant cell tumor” OR “giant cell tumour” OR “giant cell tumor of bone” OR “GCTB”) AND (“sacrum” OR “sacral” OR “spine” OR “axial skeleton”) AND (“surgery” OR “sacrectomy” OR “intralesional excision” OR “nerve-sparing surgery” OR “denosumab” OR “embolization” OR “selective arterial embolization” OR “radiotherapy” OR “recurrence” OR “pulmonary metastasis” OR “malignant transformation” OR “classification” OR “surveillance”). This strategy was applied in PubMed/MEDLINE, and the same core terms were used in Google Scholar with simplified keyword combinations to account for differences in search functionality between databases.

Reference lists of relevant articles were manually screened to identify additional studies. Articles were included if they addressed sacral GCTB specifically or provided clinically relevant data on GCTB diagnosis, classification, management, recurrence, metastasis, malignant transformation, systemic therapy, surveillance, or functional outcomes. Priority was given to peer-reviewed original studies, sacral-specific case series, larger retrospective cohorts, systematic reviews, clinically informative review articles, and selected case reports describing uncommon presentations, rare complications, or management scenarios not adequately represented in larger studies.

Articles were excluded if they were not relevant to GCTB, did not address sacral or axial disease or a directly applicable management issue, lacked sufficient clinical detail, were not available in English or English translation, or consisted of non-peer-reviewed commentary without extractable clinical information. Because this was designed as a narrative rather than a systematic review, formal PRISMA-style screening counts were not prospectively maintained. Because of the marked heterogeneity of the available evidence, including variation in tumor extent, treatment approach, outcomes, and follow-up duration, the findings were synthesized narratively rather than quantitatively.

## 3. Clinical Presentation

Sacral GCTB predominantly affects young adults and shows a slight female predominance. In the reported sacral GCTB studies, the mean age ranged from 29.5 to 34.4 years. Jamshidi et al. reported a mean age of 29.47 ± 8.14 years, with 13 females among 19 patients [6]. Martin and McCarthy reported a mean age of 31 years (range 13–49), with equal sex distribution in 10 patients [2]. Wang et al. reported a mean age of 34.4 ± 11.6 years among 205 patients, 79.5% of whom were aged 21–50 years, with 110 females and 95 males [3] [Table 1].

Pain is the most common presenting symptom and is typically described as progressive low back pain that may radiate to the buttocks, thighs, or lower limbs. In published series, pain was present in nearly all patients at diagnosis, including 10/10 patients in one cohort and 18/19 in another [2,6]. Some patients also present with a palpable mass or bowel and bladder symptoms. Symptom duration before diagnosis ranges from weeks to years; in one series, the average duration of pain before diagnosis was 30 months, highlighting the indolent course and frequent diagnostic delay of sacral GCTB lesions [2,5,6,8]. 

Neurological deficits are common at presentation and usually reflect compression or involvement of sacral nerve roots. Reported neurological symptom rates were 70% (7/10) in one series and 56% (5/9) in another, with manifestations including sensory disturbance, weakness, bowel and bladder dysfunction, and sexual symptoms; cauda equina syndrome was uncommon but documented [2,8]. Other series have also described buttock or lower-limb paresthesia and occasional cauda equina presentations [6]. Case reports of paraplegia with vesicorectal dysfunction in large tumors further illustrate that sacral GCTB may remain unrecognized until advanced disease develops, because early symptoms are often insidious and may mimic common causes of low back pain [4,9]. 

Tumor level within the sacrum strongly influences neurological risk and surgical decision-making. In the large cohort reported by Wang et al., 39.0% of sacral GCTB lesions were centered at S1–S2 and 55.6% involved both upper and lower sacral regions, whereas only 5.4% were confined to S3 or below [3]. Other series similarly describe predominant involvement at or above S2 and large tumor size; in one cohort, tumor size correlated significantly with recurrence (r = 0.654, *p* = 0.001) [6,8]. Functional outcome after sacrectomy is closely related to nerve-root preservation, with pooled data showing better ambulation and markedly improved bladder and bowel function when the S2–S3 roots, particularly bilateral S3 roots, are preserved [10]. Consistent with this, one clinical series reported no urinary or bowel dysfunction when both S3 nerves were preserved following conservative surgery [11] [Table 2]. Overall, these findings suggest that larger tumors with cephalad extension above S2 are more likely to produce neurological compromise, whereas preservation of bilateral S3 roots remains a key determinant of postoperative continence and functional recovery [6,10,11].

## 4. Diagnostic Approach

### 4.1. Imaging Modalities

Imaging is central to the diagnosis and preoperative assessment of sacral GCTB lesions. On plain radiographs, sacral GCTB typically appears as an expansile lytic lesion with geographic bone destruction and little or no periosteal reaction unless a pathological fracture is present; a soap-bubble appearance may occasionally be seen [12]. In the sacrum, these tumors are commonly eccentric, often involve the upper sacrum, and may abut or extend across the sacroiliac joint. Their purely lytic appearance and absence of matrix calcification may help distinguish them from other sacral tumors [12,13].

Computed tomography is useful for defining cortical destruction, intraosseous extent, extraosseous extension, and the relationship of the tumor to adjacent osseous and pelvic structures. Three-dimensional reconstruction may assist operative planning [12]. Comparative imaging studies further show that sacral GCTB lesions are commonly eccentric lesions of the upper sacrum, with predominant intraosseous expansion and frequent internal cystic change, including occasional fluid–fluid levels, which may assist in distinguishing them from other sacral tumors [14].

Contrast-enhanced magnetic resonance imaging (MRI) is the preferred modality for evaluating sacral GCTB because it best delineates soft-tissue extension, sacral canal involvement, epidural spread, and compression of adjacent nerve roots [4,12,15]. Typical findings include low T1 signal and heterogeneous low-to-intermediate T2 signal, with low signal intensity often attributed to hemosiderin, hemorrhagic change, or fibrosis [12,13,15]. This relatively low T2 signal may help differentiate sacral GCTB from many other spinal tumors, which are more often hyperintense on T2-weighted imaging [15]. MRI is also the most useful modality for postoperative surveillance and detection of residual or recurrent disease [13].

Chest computed tomography (CT) should be done as part of systemic staging to rule out pulmonary metastasis, which is the most common site of metastasis in GCTB. The role of positron emission tomography (PET) remains less well defined, but 18F-fluorodeoxyglucose (18F-FDG) PET/CT may help assess tumor metabolism, with higher uptake in solid components and lower uptake in cystic or aneurysmal areas. Increased FDG uptake has also been described in malignant sacral GCTB [4,12,16]. At present, PET is best regarded as an adjunctive rather than a routine diagnostic tool.

Although imaging, particularly MRI, plays a central role in recognizing the characteristic pattern of sacral GCTB, defining local extent, and assessing soft-tissue and neural involvement for treatment planning, definitive diagnosis still depends on tissue confirmation because of radiologic overlap with other sacral tumors.

### 4.2. Biopsy and Pathologic Confirmation

Histopathologic confirmation is essential in suspected sacral GCTB because imaging alone is not diagnostic. CT-guided core needle biopsy is commonly used and typically demonstrates multinucleated osteoclast-like giant cells within a mononuclear stromal background; secondary aneurysmal bone cyst changes may also be present [4,17]. CT-guided needle biopsy has been reported to provide diagnostic tissue in all attempted sacral GCT cases in one series. Because biopsy findings may affect definitive surgical planning, the biopsy route should be planned in coordination with the treating surgeon to minimize neurovascular risk and preserve subsequent treatment options [2]. Molecular testing can support the diagnosis when tissue is limited: H3F3A p.Gly34 mutations, particularly p.G34W, are present in the vast majority of GCTB cases and are highly supportive of the diagnosis, whereas immunohistochemistry for the H3.3 G34W mutant protein provides a practical confirmatory tool by showing strong nuclear staining in neoplastic stromal cells with negative giant cells [17,18]. These markers are particularly useful when differentiating GCT from histologic mimics.

### 4.3. Differential Diagnosis

The differential diagnosis of sacral GCTB includes both benign and malignant lesions. Chordoma is an especially important consideration because it more often presents as a central midline lesion in the lower sacrum and typically affects older patients, whereas sacral GCTB more often shows eccentric upper sacral involvement [14]. Giant sacral schwannoma may also be eccentric but more often contains larger central cystic regions [14]. Aneurysmal bone cyst may closely mimic GCTB radiologically, particularly when fluid–fluid levels are present, but molecular testing can help distinguish primary ABC, which is associated with USP6 rearrangements, from GCTB, which harbors H3F3A mutations [13,17]. Metastasis, malignant GCTB, multiple myeloma, lymphoma, primary bone sarcoma, and other sacral tumors should also be considered [13]. Advanced MRI-based radiomics models have shown moderate performance in distinguishing sacral chordoma, sacral GCTB, and sacral metastasis, and may serve as an adjunct in difficult cases [19].

## 5. Classification and Staging

**Figure 1:**
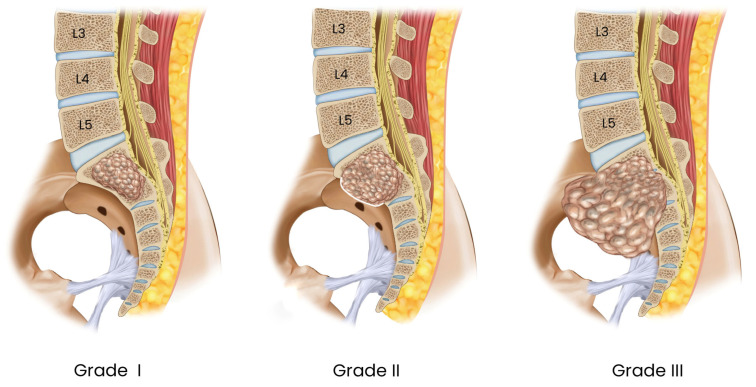
Campanacci classification of giant cell tumor of the sacrum. Grade I: The tumor has a clear, distinct border, and the overlying cortex is completely intact. Grade II: The tumor often makes the cortex thinned and expanded. Grade III: The tumor is characterized by cortical destruction and extension of the tumor into the surrounding soft tissue.

Classification of GCTB has traditionally relied on radiographic and histopathologic systems, most commonly the Enneking and Campanacci classifications [20,21] [Table 3]. The Enneking system categorizes benign musculoskeletal tumors as latent (Stage 1), active (Stage 2), or aggressive (Stage 3), whereas the Campanacci system grades lesions according to cortical integrity, margin definition, and soft-tissue extension [Figure 1] [20,21]. Because sacral GCTB lesions frequently present with cortical destruction, extraosseous extension, and locally aggressive behavior, many are functionally consistent with Enneking Stage 3 and Campanacci Grade III at diagnosis [6,22].

Although these systems broadly reflect tumor aggressiveness, their utility in sacral GCTB is limited. Neither Enneking nor Campanacci adequately captures epidural extension, sacral nerve-root involvement, expected neurological loss, bowel or bladder consequences, or the need for spinopelvic reconstruction, all of which are central to treatment planning in the sacrum [21,23]. In addition, the prognostic value of Campanacci grading remains inconsistent, because radiographic grade alone does not reliably predict recurrence, postoperative function, or the optimal surgical strategy in all patients [22,23].

For sacral tumors, anatomical classification based on vertical level, extent of sacral involvement, and anticipated nerve-root sacrifice is often more clinically informative than conventional radiographic grading alone [24]. Higher sacral involvement is associated with greater neurological morbidity, more complex operative exposure, and a higher likelihood of requiring stabilization or reconstruction [24]. Consistent with this, Wang et al. reported that most sacral GCTB lesions involve the upper sacrum, with 39.0% centered at S1–S2 and 55.6% extending across both upper and lower sacral regions, whereas only 5.4% were confined below S3 [3]. Accordingly, in sacral GCTB, anatomical extent and expected functional morbidity should be regarded as core classification considerations when planning treatment.

## 6. Multidisciplinary Management Approach

### 6.1. Individualization of Management Strategy

There is no universally accepted treatment algorithm for sacral GCTB. Management should be individualized through multidisciplinary decision-making involving orthopedic oncology, spine surgery, radiology, pathology, radiation oncology, and interventional radiology when needed. Treatment selection depends mainly on tumor size, cranial extent within the sacrum, extraosseous and pelvic extension, expected neurological morbidity, and the need to preserve lumbopelvic stability and bowel, bladder, and sexual function [25,26]. Across available series, the central therapeutic challenge in sacral GCTB is to maximize local control while minimizing treatment-related functional loss.

### 6.2. Surgical Management

Surgery remains the principal treatment for sacral GCTB, especially among patients with resectable tumors, progressive neurological compromise, or a need for sustained local control. The most commonly reported surgical management option is intralesional, nerve-sparing excision, which aims to reduce tumor burden while preserving sacral nerve root function if feasible [11,25,27]. In a long-term series from Memorial Sloan Kettering Cancer Center, Domovitov et al. described 24 patients treated with intralesional procedures while preserving at least the S1–S3 nerve roots, most through a posterior approach [25]. Guo et al. similarly reported conservative surgery in 24 patients, with preservation of bowel and urinary function, particularly when both S3 roots were spared [11]. These findings support intralesional nerve-sparing surgery as a pragmatic option when functional preservation is prioritized, although recurrence risk remains substantial [11,25,27].

En bloc resection may provide better oncologic control in certain cases, yet it is limited by the close proximity of sacral GCTB to sacral nerve roots and nearby neurovascular structures, along with pelvic visceral organs. Thus, wider resection is often linked with greater postoperative neurological morbidity, especially in the upper sacrum [24,28,29]. Therefore, en bloc resection is better reserved for specifically selected cases where the gains in local control outweigh the burden of functional sacrifice and sacral reconstruction. Recent reports suggest that reconstruction with custom implants and nerve reconstruction may be feasible in highly selected cases, but evidence remains limited [28]. 

The operative approach depends largely on tumor level and anterior extension. Lesions extending above S2 often require combined anterior–posterior exposure, whereas tumors confined below S3 may be managed via a posterior-only approach if pelvic organ or major vascular involvement is absent [25,29]. Reconstruction and spinopelvic stabilization are considered when resection compromises sacroiliac support or a substantial portion of S1. In the Memorial Sloan Kettering series, spinopelvic fusion was performed in 8 of 24 patients, and long-term stability was maintained in nearly all cases [25].

Surgical morbidity remains considerable. Reported complications include wound infection, major blood loss, cerebrospinal fluid leakage, and postoperative neurological dysfunction [25,27]. Domovitov et al. reported wound infection in 29% of patients and temporary complete loss of bladder and/or bowel function in 12.5%, although urinary control was eventually regained in affected patients; larger tumor volume was associated with greater postoperative neurological loss [25]. Across reported series, infection rates after nerve-sparing surgery range from 10% to 36%, underscoring the complexity of operative management in this region [27].

### 6.3. Denosumab

Denosumab is a monoclonal antibody against RANKL that suppresses osteoclast-mediated bone resorption and is used in GCTB that is unresectable or likely to require surgery with severe functional loss [26,27,30,31]. In sacral GCTB, it is particularly relevant for patients in whom resection would be expected to cause major neurological morbidity, for patients requiring downstaging before surgery, and for selected patients in whom long-term disease control rather than cure is the main objective [26,27,30,31]. Phase II studies in GCTB have shown that denosumab can induce tumor response, reduce giant-cell burden, promote ossification, and, in some patients, defer or avoid surgery altogether [30,31]. Despite these benefits, several clinically important controversies remain unresolved. The optimal duration of denosumab therapy, the role of maintenance or dose-spacing regimens, and the safest discontinuation strategy have not been standardized, particularly in sacral disease, where non-surgical treatment may require prolonged therapy [26,27,32]. Discontinuation may be followed by disease reactivation, and rebound hypercalcemia has been reported, especially in younger patients and after prolonged treatment; therefore, cessation should be planned carefully with biochemical and radiologic monitoring [26,32,33]. In patients treated non-operatively, denosumab should be understood primarily as a disease-control strategy rather than a curative treatment, and long-term surveillance remains necessary [26,27,32].

The timing of denosumab before surgery is also controversial. Preoperative denosumab may reduce pain, vascularity, and soft-tissue extension and may facilitate less morbid surgery in selected high-risk cases [26,27,30,31]. However, treatment-induced sclerosis and peripheral ossification may obscure residual tumor, make curettage more difficult, and potentially increase the risk of local recurrence after intralesional surgery [26,27,32]. In the sacral series summarized by Tsukamoto et al., recurrence occurred in 2 of 3 patients who received preoperative denosumab compared with 2 of 6 who did not, although the small sample size and selection bias limit interpretation [27]. Therefore, when intralesional surgery is planned, denosumab should be used selectively and with a predefined surgical strategy rather than routinely.

Reported adverse effects include hypophosphatemia, osteonecrosis of the jaw, anemia, atypical femoral fracture, and rebound hypercalcemia after discontinuation [26,27,32,33]. Denosumab is contraindicated during pregnancy because of potential fetal toxicity [27,34]. Malignant transformation during or after denosumab therapy has been reported, but causality remains uncertain because conventional GCTB may rarely transform spontaneously and because denosumab-induced histologic changes may mimic sarcomatous features [26,32,35]. Overall, denosumab appears most useful when the primary aim is to control unresectable disease, reduce the morbidity of surgery, or avoid function-sacrificing resection, but its duration, perioperative timing, and discontinuation strategy should be individualized.

### 6.4. Radiotherapy

Radiotherapy has been used as primary treatment, adjuvant therapy, or salvage treatment for unresectable sacral GCTB, residual disease, or recurrent disease when surgery would carry unacceptable morbidity [25,36,37]. Its role remains debated because sacral GCTB often affects young patients, and historical concerns regarding radiation-induced sarcoma have limited its routine use.

It is important to distinguish older radiotherapy techniques from modern treatment approaches. Earlier reports of malignant transformation were often associated with older orthovoltage techniques or less conformal radiation delivery, whereas more recent megavoltage and conformal techniques appear to carry a lower risk of radiation-induced malignancy while providing local control in selected difficult cases [36,37]. In one clinical series, preoperative radiotherapy at doses of 40–66 Gy was associated with improved disease-free survival and no radiation-induced sarcoma [25]. In contrast, Ruggieri et al. reported one death from high-grade sarcoma in a patient who had received radiotherapy, highlighting that the risk, although uncommon, remains clinically important [38].

Accordingly, radiotherapy should not be considered routine first-line treatment for resectable conventional sacral GCTB. It may be considered in selected patients with unresectable, residual, recurrent, or refractory disease when surgery is not feasible, when expected surgical morbidity is excessive, or when denosumab is unavailable, contraindicated, ineffective, or unsuitable for long-term use. Decisions regarding radiotherapy should be individualized through multidisciplinary discussion, balancing expected local control against patient age, tumor extent, prior treatments, risk of malignant transformation, wound complications, fertility concerns, and availability of alternative therapies such as denosumab or selective arterial embolization.

### 6.5. Selective Arterial Embolization

Selective arterial embolization has two main roles in sacral GCTB: as a preoperative adjunct and as a primary or repeated treatment in selected unresectable cases [25,39,40]. As a preoperative adjunct, embolization may reduce vascularity, facilitate resection, and improve oncologic outcomes; Domovitov et al. reported that embolization performed one day before surgery was associated with lower recurrence risk [25]. As a primary treatment, repeated embolization has shown durable symptom relief and radiographic control in selected patients, with generally low rates of permanent bowel, bladder, or sexual dysfunction attributable to the procedure [39,40]. Outcomes appear more favorable in primary than recurrent tumors [39].

Combined strategies using serial embolization with denosumab have also been reported for large sacropelvic tumors and may offer benefit in selected patients, although evidence remains limited [41]. Because different embolic agents and treatment intervals have been used across studies, the optimal protocol remains undefined [39]. Thus, embolization is best viewed as a flexible adjunct or alternative for carefully selected patients rather than a standardized standalone pathway.

### 6.6. Practical Synthesis

Taken together, current evidence suggests that management of sacral GCTB should be individualized, taking into consideration tumor extent, the balance between oncologic control and functional preservation, and minimizing morbidity. Intralesional nerve-sparing surgery remains a practical option for many patients with resectable disease, particularly when sacral nerve-root preservation and spinopelvic stability can be maintained. En bloc resection may offer stronger local control in selected distal or surgically favorable tumors, but in upper sacral disease, it often carries substantially greater neurological and reconstructive morbidity. Denosumab, selective arterial embolization, and radiotherapy may serve as adjunctive or alternative modalities in selected patients with unresectable disease, high-morbidity surgical scenarios, residual disease, or recurrent disease.

However, the available evidence is derived mainly from retrospective series, small cohorts, heterogeneous case series, and selected case reports. Therefore, recurrence rates, complication profiles, and functional outcomes reported for surgery, denosumab, selective arterial embolization, and radiotherapy should not be interpreted as directly comparable across modalities. Differences in tumor extent, primary versus recurrent presentation, treatment indication, follow-up duration, and outcome definitions limit direct comparison. Accordingly, the evidence summarized in Table 4 should be viewed as a structured clinical synthesis rather than a comparative ranking of treatment efficacy.

Treatment goals should also be distinguished according to disease context. In primary resectable disease, the goal is durable local control while preserving sacral nerve-root function, bowel and bladder function, and spinopelvic stability whenever feasible. In recurrent disease, management should focus on confirming recurrence, reassessing histology when behavior is atypical, evaluating pulmonary metastatic risk, and minimizing cumulative treatment morbidity. In unresectable disease, the goal is disease stabilization, symptom control, and preservation of function. In high-morbidity resectable disease, denosumab and/or selective arterial embolization may be considered to avoid, delay, or reduce the morbidity of surgery, while radiotherapy is generally reserved for selected refractory, residual, or non-operable cases.

**Figure 2:**
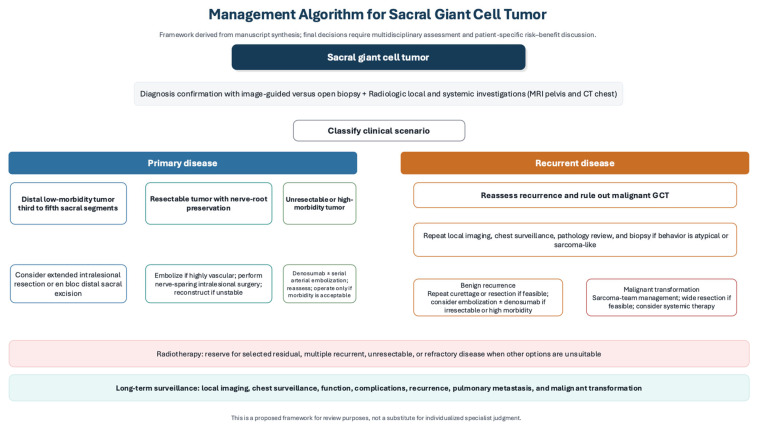
Management Algorithm for Sacral Giant Cell Tumor.

Given the anatomical complexity and heterogeneity of sacral giant cell tumor of bone and the absence of standardized management guidelines, treatment selection should be individualized through multidisciplinary discussion. Previous authors have proposed treatment strategies for selected scenarios; for example, Puri et al. described a non-operative, function-preserving approach using short-term denosumab, angioembolization, and radiotherapy in different combinations for sacral tumors in which surgery could endanger important neural structures [44]. Building on the best available literature, we propose a broader practical management algorithm in Figure 2 that integrates disease status, tumor level, neurological involvement, resectability, anticipated morbidity, spinopelvic stability, and the potential roles of surgery, denosumab, selective arterial embolization, radiotherapy, and surveillance. This algorithm is intended as a flexible clinical framework rather than a rigid protocol or direct comparative hierarchy, because treatment decisions must account for tumor extent, recurrence status, expected morbidity, institutional expertise, patient factors, and patient preferences.

## 7. Follow-Up and Surveillance

Surveillance strategies specific to sacral GCTB have not been standardized, and available recommendations are based mainly on extrapolation from broader GCTB practice, retrospective sacral series, and expert opinion [42,45,46,47]. Therefore, follow-up should be individualized according to recurrence risk, treatment modality, residual disease status, and metastatic risk. As a practical approach, patients may undergo clinical assessment and local imaging every 3 months during the first 2 years, every 6 months during years 3–5, and annually thereafter in selected patients. Prolonged follow-up beyond 5 years is reasonable, particularly in patients with large tumors, residual disease, recurrent disease, non-surgical disease control, or prior denosumab or radiotherapy exposure.

Chest surveillance is an important component of follow-up because pulmonary metastasis, although uncommon, is a recognized event in GCTB and appears to be strongly associated with local recurrence. However, the optimal chest imaging modality and interval remain uncertain. A risk-adapted strategy is therefore reasonable. Baseline chest CT may be obtained for systemic staging, while follow-up surveillance may use chest radiographs or low-dose chest CT, depending on risk. Chest radiographs may be reasonable for lower-risk patients with completely treated primary disease and no recurrence, whereas chest CT is preferred for patients with recurrent disease, residual or progressive disease, pulmonary symptoms, suspicious radiographic findings, or aggressive clinical behavior. Fellows et al. demonstrated substantial heterogeneity in thoracic surveillance practices across sarcoma centers and reported a pulmonary metastasis rate of 9.6%, with local recurrence emerging as the only independent predictor of metastatic disease [46]. In patients with known pulmonary nodules or metastatic disease managed initially with observation, CT-based monitoring is preferred because nodule size and progression influence prognosis [48].

MRI is the preferred modality for detecting local recurrence because of its superior soft-tissue contrast and ability to evaluate the postoperative bed [12,42]. CT may be used as a complementary modality when MRI is contraindicated or when cortical integrity, ossification, reconstruction, or spinopelvic stability requires assessment. Findings suggestive of recurrence include a focal rounded or mass-like postoperative lesion with high T2 signal and enhancement, while progressive focal osteolysis on serial radiographs or CT may provide an additional clue [12,42,49]. Although most recurrences are reported within the first 12–18 months after surgery, late recurrence is well documented; therefore, surveillance should combine closer early assessment with long-term annual review in selected patients [6,27,47,49].

Long-term follow-up should also include functional assessment, particularly in patients who have undergone surgery involving sacral root manipulation or resection. The Musculoskeletal Tumor Society (MSTS) score remains one of the most widely reported instruments for postoperative functional evaluation and captures pain, function, and emotional acceptance [6,47]. However, in sacral GCTB, follow-up should also explicitly address bowel, bladder, sexual, gait, and pain-related outcomes, as these domains may change over time and are central to treatment burden and quality of life. Overall, surveillance in sacral GCTB should be viewed not only as recurrence detection, but also as an ongoing multidisciplinary process of oncologic, neurological, and functional monitoring.

## 8. Outcomes and Prognosis

Outcomes in sacral GCTB lesions vary substantially according to tumor extent, treatment modality, and the degree of neurological preservation achieved. Across reported series, local control after treatment remains variable, reflecting the central challenge of sacral GCTB management: more aggressive local therapy may improve tumor control, but often at the cost of greater functional morbidity [6,27,38,47]. Intralesional surgery can provide durable disease control in selected patients, yet recurrence remains common, with some series reporting rates exceeding 40–50% [6,27,38,47].

Time-dependent recurrence outcomes are inconsistently reported across sacral GCTB series. Guo et al. reported recurrence in 7 of 24 patients (29.2%) after conservative surgery, with a mean time to first recurrence of 13 months and a 5-year local recurrence-free survival rate of 69.6% [11]. Other sacral series more commonly report crude recurrence rates rather than standardized 5- or 10-year progression-free survival, with recurrence rates ranging from 22% to 42.1% across the series summarized in Table 1 [2,6,8]. Therefore, available evidence suggests that recurrence is common, but direct comparison of 5- and 10-year PFS across modalities remains limited by inconsistent outcome reporting and heterogeneous follow-up and the scarcity of the literature due to the rare occurrence of such anatomic locations.

Larger tumor size has also been associated with increased recurrence risk [6]. By contrast, serial selective arterial embolization has shown durable long-term control in selected patients, whereas denosumab-based and other non-surgical strategies may stabilize disease and prevent radiologic progression without necessarily achieving disease-free status [27,39,43].

Neurological and functional outcomes are closely linked to the extent of resection and preservation of sacral nerve roots. Ruggieri et al. reported that major L5-S2 deficits decreased after intralesional decompression, whereas minor S3-S4 deficits became more common, highlighting the vulnerability of distal sacral roots during surgery [38]. Functional series likewise emphasize the value of nerve preservation: Jamshidi et al. reported a mean MSTS score of 74.7 ± 16.78, while van der Heijden et al. found significantly better MSTS scores in patients without postoperative complications [6,47]. Tsukamoto et al. also noted better bowel, bladder, and motor outcomes in non-surgically managed patients than in those undergoing intralesional surgery, consistent with the lower risk of sphincter and motor dysfunction when nerve root sacrifice is avoided [27]. Overall, these findings indicate that postoperative function depends not only on tumor control, but also on the neurological cost of achieving that control.

Treatment-related morbidity remains substantial, particularly after surgery. Reported complications include wound infection, major intraoperative bleeding, and postoperative neurological dysfunction [25,38]. Ruggieri et al. documented complications in 48% of patients, including wound problems, hemodynamically significant bleeding, and two deaths related to pulmonary embolism and radiation-induced sarcoma [38]. Domovitov et al. likewise reported wound infection in 29% and temporary complete loss of bladder and/or bowel function in 12.5% of patients, although urinary control was eventually regained in the affected cases [25]. These findings underscore that treatment success in sacral GCTB cannot be judged solely by recurrence rates, but must also account for perioperative morbidity and long-term functional burden.

Pulmonary metastasis is uncommon but clinically important. Ebeid et al. reported lung metastases in 3.2% of 466 patients with GCTB, occurring exclusively in patients with prior local recurrence, while other series have reported overall metastatic rates ranging from 1% to 9% [46,50,51]. Local recurrence has consistently emerged as the strongest predictor of metastatic spread [46,51]. In patients managed initially with observation, progression may occur within months, and nodules larger than 5 mm have been associated with worse progression-free survival [48]. Overall, current evidence suggests that prognosis in sacral GCTB is determined not only by oncologic control, but also by preservation of neurological function, treatment-related morbidity, and the risk of recurrence-driven metastatic disease.

## 9. Special Considerations

### 9.1. Pediatric and Adolescent Patients

Pediatric giant cell tumor of bone is uncommon, and sacral involvement is particularly rare. In a large single-institution series, only 15 of 910 GCTB cases (1.6%) occurred in patients younger than 16 years, with one sacral case managed with denosumab alone and remaining alive with disease at 96 months [52]. In this setting, H3F3A/H3.3 G34W testing is especially useful for confirming the diagnosis and distinguishing GCTB from other osteoclast-rich lesions in younger patients [52]. Denosumab may be effective in unresectable pediatric sacral GCTB, but its use in growing children remains cautious because of potential concerns of bone modeling, sclerosis near growth plates, and the risk of severe rebound hypercalcemia after discontinuation [33].

### 9.2. Pregnancy and Sacral GCTB

Pregnancy-associated GCTB may demonstrate accelerated growth and therefore requires close monitoring. In a retrospective series of women with GCTB, pregnancy-associated tumors showed significant interval growth, and two cases involved the sacrum [34]. Management is challenging because denosumab is contraindicated during pregnancy as it has potential fetal toxicity. As a result, treatment is limited to carefully selected surgery, catheter embolization in appropriate pelvic or sacral lesions, serial imaging surveillance, and supportive care, with management individualized according to gestational timing, symptoms, and tumor behavior [34]. Denosumab and other treatment modalities could be considered after delivery.

### 9.3. Recurrence and Pulmonary Metastatic Risk

Recurrent sacral GCTB requires careful reassessment because repeated treatment may increase cumulative morbidity, while local recurrence is also associated with a higher risk of pulmonary metastasis. This is particularly relevant in sacral disease, where delayed diagnosis, large tumor burden, proximity to sacral nerve roots, and anatomical constraints may limit complete excision and complicate the interpretation of recurrence [6,25,38]. Therefore, recurrent disease should not be evaluated only as a local oncologic event, but also as a marker of increased metastatic risk and treatment-related functional burden.

In patients with recurrent or progressive disease, reassessment should include updated local imaging, chest surveillance, review of the original histopathology, and consideration of repeat biopsy when the clinical or radiologic behavior is atypical. Rapidly progressive recurrence, unexpected soft-tissue growth, aggressive cortical destruction, or discordance between imaging findings and the initial histologic diagnosis should prompt repeat biopsy and multidisciplinary sarcoma-team reassessment [17,35,53].

### 9.4. Malignant Transformation and Atypically Aggressive Behavior

Malignant transformation of GCTB is rare but clinically important. In a pooled review of 2315 patients, the overall malignancy rate was 4.0%, including 1.6% primary and 2.4% secondary malignant GCTB; approximately 75% of secondary cases occurred after radiotherapy, and outcomes were substantially worse than for primary malignant disease [53]. Malignant transformation has also been reported after denosumab therapy, sometimes with short latency, although a causal relationship remains uncertain and requires further study [35]. Importantly, spontaneous malignant transformation in previously untreated GCTB has also been described, suggesting that secondary malignancy may in some cases reflect the natural history of the tumor rather than treatment effect alone [54].

Although conventional GCTB is classified as a benign but locally aggressive tumor, a small subset may demonstrate atypically aggressive behavior that clinically resembles sarcoma, including rapid local recurrence, extensive soft-tissue progression, pulmonary metastasis, or malignant transformation [35,53,54]. Reported malignant transformation may occur as primary malignant GCTB, secondary transformation after prior treatment, or spontaneous transformation in previously untreated tumors [53,54]. Denosumab-associated malignant transformation has also been reported, although causality remains uncertain and treatment-induced histologic changes may mimic sarcomatous features [26,32,35]. These findings support careful histopathologic evaluation and close long-term surveillance, particularly in recurrent disease and in patients previously treated with radiotherapy or denosumab [35,53,54].

### 9.5. Role of Chemotherapy in Malignant GCTB

Conventional aggressive or recurrent GCTB should be distinguished from malignant GCTB or sarcomatous transformation, because the role of chemotherapy differs substantially between these entities. For conventional sacral GCTB, available treatment data mainly support surgery, denosumab, selective arterial embolization, radiotherapy in selected cases, and surveillance rather than sarcoma-type chemotherapy [26,37,39]. Therefore, chemotherapy should not be considered routine for aggressive benign GCTB.

In contrast, when malignant transformation is confirmed histologically, treatment is generally individualized according to high-grade bone sarcoma principles, including wide resection when feasible and consideration of systemic chemotherapy based on the sarcomatous component, most commonly osteosarcoma-like differentiation [26,35,53]. Thus, chemotherapy may be relevant when repeat biopsy confirms malignant GCTB or another high-grade sarcoma arising in association with GCTB, but current evidence remains limited and should be interpreted cautiously [26,35,53].

## 10. Future Directions

Future research in sacral giant cell tumor should focus on improving risk stratification, refining systemic therapy, and reducing treatment-related morbidity. Although denosumab can induce meaningful clinical and radiologic responses and facilitate surgical downstaging, important uncertainties remain regarding optimal treatment duration, maintenance dosing, long-term safety, recurrence risk after curettage, and its possible association with malignant transformation. Comparative evidence for alternatives such as zoledronic acid and combination regimens also remains limited [26,32].

Molecular profiling is increasingly relevant to both diagnosis and therapeutic development. H3F3A p.Gly34 mutations are highly prevalent in GCTB and serve as an important diagnostic marker, particularly in limited biopsy samples [17]. Additional alterations involving epigenetic regulators suggest broader biological complexity, while malignant H3F3A-wildtype tumors may harbor actionable fusions such as BRAF- or ALK-related rearrangements, supporting the future potential of genomically guided therapy [55,56].

Advances in sacral reconstruction and surgical assistance may also expand treatment options. Early reports of custom and modular 3D-printed sacral implants have shown encouraging osseointegration and functional outcomes after nerve-sparing resection, while robot-assisted sacral tumor surgery appears technically feasible in selected benign lesions [57,58,59]. However, these approaches remain supported by limited early experience and require validation in larger comparative studies. Overall, collaborative registries and prospective multicenter studies are needed to better define optimal indications, long-term outcomes, and individualized treatment strategies in sacral GCTB [32].

## 11. Limitations

This review has several limitations. First, the available literature on sacral giant cell tumor is scarce by the rarity of the disease, with most evidence derived from retrospective series, small cohorts, and selected case reports. Second, substantial heterogeneity in tumor extent, treatment strategies, outcome definitions, and follow-up duration limits direct comparison across studies. Third, much of the published evidence combines sacral tumors with spinal or pelvic giant cell tumors, which may reduce the specificity of conclusions for sacral disease alone. Finally, because of the lack of randomized or prospective comparative studies, many management recommendations remain based on expert opinion, institutional experience and extrapolation from giant cell tumor of bone at other anatomical sites.

## 12. Conclusions

Sacral GCTB is a rare, yet locally aggressive neoplasm whose management is determined by its unique anatomical, neurological, and reconstructive challenges faced in the sacrum. Delayed presentation is common, and treatment approaches should balance between local tumor control and preservation of sacral nerve root function, bowel, bladder, and sexual function, along with lumbopelvic stability. The mainstay for sacral GCTB treatment remains surgery for resectable tumors. However, denosumab, selective arterial embolization, and radiotherapy each play an important role in selected patients, particularly in non-operable cases or cases carrying substantial morbidity. Since recurrence, pulmonary metastasis, and treatment-related functional burden may emerge late, long-term surveillance should be considered an integral component of care. Overall, the current evidence supports a multidisciplinary, individualized approach taking into consideration the anatomical extent, expected neurological deficit and cost, and patient-centered functional outcomes to guide the appropriate treatment selection. Further prospective multicenter studies are needed to refine treatment algorithms, clarify the long-term role of systemic therapy, and improve risk stratification in sacral giant cell tumors.

## Figures and Tables

**Table 1:** Demographics and clinical presentation of sacral GCTB.

Study	N	Mean Age (Range)	Female %	Most Common Symptoms	Neurological Deficit at Presentation	Mean Tumor Size	Symptoms Duration Before Diagnosis
Martin & McCarthy (2010) [2]	10	31 yrs (13–49)	50%	Low back pain	70% (7/10)	Not reported	30 months (1 wk–10 yrs)
Thangaraj et al. (2010) [8]	9	34 yrs (15–52)	67%	Back/buttock pain	56% (5/9)	10 cm	4 weeks–1 year
Jamshidi et al. (2017) [6]	19	29.5 yrs (18–46)	68.4%	Pain	26% (5/19)	6.3 cm (2–15)	Not specified
Wang et al. (2020) [3]	205	34.4 yrs (11–67)	53.7%	Not detailed	Not detailed	Not detailed	Not detailed

**Table 2:** Expected residual neurological function after sacral nerve-root resection during en bloc sacrectomy [10].

Level of Sacral Root Preservation	Normal Ambulation	Normal Bladder Continence	Normal Bowel Continence
Bilateral S3 + at least one S4 preserved	100%	100%	100%
Bilateral S3 preserved (S4–S5 sacrificed)	94.1%	83.3%	94.0%
Bilateral S2 preserved (S3–S5 sacrificed)	56.2%	39.9%	50.0%
Unilateral sacral root resection	Preserved	75.0%	82.6%
Both S2 sacrificed	At risk	0%	0%

Note: These data are derived from the sacrectomy and sacral nerve-root resection literature and are most applicable when considering en bloc sacrectomy or anticipated nerve-root sacrifice. They should not be generalized to all sacral GCTB treatment scenarios, including intralesional nerve-sparing surgery, denosumab, selective arterial embolization, or surveillance-based management.

**Table 3:** Classification systems relevant to sacral GCTB and their clinical limitations.

System	Basis	Categories	Application to Sacral GCTB	Limitations in Sacral GCTB	Reference
Enneking (Benign)	Radiographic tumor–host margin	Stage 1 (Latent): well-demarcated; Stage 2 (Active): indistinct borders; Stage 3 (Aggressive): soft tissue extension	Most sacral GCTB lesions classified as Stage 3 (aggressive) due to cortical breach and extraosseous extension	Does not account for epidural space, nerve root sacrifice, or spinal stability	[20,21]
Campanacci	Radiographic appearance	Grade I: intact cortex, sclerotic rim; Grade II: thinned cortex, no rim; Grade III: cortical breach, soft tissue mass	Majority of sacral GCTB lesions present as Grade III (68.4% in Jamshidi et al.)	Prognostic value for recurrence debated; may not reliably predict outcomes	[6,20,22,23]
Fourney Sacrectomy Classification	Radiographic appearance	Low (below S3); Middle (S2–S3); High (S1–S2); Total (above S1)	Directly guides surgical approach and predicts functional morbidity	Descriptive of surgical approach rather than tumor biology	[24]

**Table 4:** Summary of key sacral GCTB studies reporting treatment, recurrence, complications, and functional outcomes.

Study	N	Treatment	Follow-Up	Recurrence/Control	Complications/Function
Martin & McCarthy, 2010 [2]	10	Embolization + intralesional resection: 6; en bloc resection: 2; embolization only: 1; no treatment: 1	Mean 31.9 months	2 recurrences among 9 surgically treated patients; no lung metastasis	Neurologic symptoms in 7 patients at presentation; Incomplete neurologic recovery in 5 patients at final follow-up
Thangaraj et al., 2010 [8]	9	Curettage: 7; radiotherapy: 1; distal sacral excision: 1; embolization used before later curettages	2–21 years	Local recurrence in 3/7 after curettage; all later controlled with embolization, surgery, or radiotherapy	2 patients had worse neurology than presentation; 3 required spinopelvic fusion; all alive, mobile, and active
Guo et al., 2009 [11]	24	Conservative surgery with intraoperative abdominal aorta occlusion	Mean 58 months	Local recurrence in 7 patients; 5-year local recurrence-free survival 69.6%; lung metastasis in 2 patients	Complications in 10 patients; wound complications in 7; abnormal urinary function in 7 and abnormal bowel function in 8
Jamshidi et al., 2017 [6]	19	Intralesional curettage: 16; marginal resection: 3	Mean 158.5 months	Local recurrence in 8 patients; all after curettage; 1 pulmonary metastasis	Infection in 5; postoperative neurologic deficit in 6; mean MSTS 74.7%
Domovitov et al., 2016 [25]	24	Intralesional surgery; radiotherapy in 14; embolization in 16; cryosurgery in 19; spinopelvic fusion in 8	Mean 87 months	Local recurrence in 7/23; distant recurrence in 3; no local recurrence with combined radiotherapy + embolization	Severe bowel/bladder dysfunction in 3, all later improved; infections in 7; spinopelvic instability in 1
Ruggieri et al., 2010 [38]	31	Intralesional surgery; radiotherapy in 21; embolization in 23; local adjuvants in 17	Median 108 months	Local recurrence in 3; survival to local recurrence 90% at 60 and 120 months; no metastases	Complications in 15; wound complications in 8; massive bleeding in 7; 2 deaths
van der Heijden et al., 2014 [42]	26	Intralesional excision in all; local adjuvants in 21; radiotherapy in 5; interferon-α in 3; bisphosphonates in 1	Median 98 months	Local recurrence in 14; 2-year recurrence-free survival 20% after isolated curettage and 65% with local adjuvants; pulmonary metastases in 4	Complications in 12; median MSTS 24; MSTS better without complications
Lin et al., 2002 [43]	18	Selective arterial embolization in all; intra-arterial cisplatin in 9	Median 105 months	Favorable response in 14; local control 57% at 10–20 years; late sacral recurrence/progression in 3	Neurologic complications in 3; no bowel, bladder, or sexual decline from embolization; 1 treatment-related death
Tsukamoto et al., 2021 [27]	15	Nerve-sparing surgery in 9; non-surgical treatment in 6 with denosumab ± embolization	Mean 85 months for surgery; 59 months for non-surgical treatment	Local recurrence in 4/9 after surgery; tumor progression in 0/6 after non-surgical treatment; lung metastasis in 1	Complications in 4/9 after surgery and 1/6 after non-surgical treatment; modified Biagini score better in non-surgical group

## Data Availability

No new data were created or analyzed in this study.
